# Psychological Barriers and Perceptual Drivers of Sensor-Based Smart Health Technology Adoption

**DOI:** 10.3390/s25227029

**Published:** 2025-11-18

**Authors:** Dat Hung Ho, Byeong-Hoon Lee, Byungkwon Jeon, Hak-Seon Kim

**Affiliations:** 1 Department of Global Business, Kyungsung University, Busan 48434, Republic of Korea; hohungdat2@ks.ac.kr; 2 Cheil Electric Co., Ltd., Busan 49437, Republic of Korea; bhlee@cheilelec.com (B.-H.L.); bkjeon@cheilelec.com (B.J.); 3 School of Hospitality & Tourism Management, Kyungsung University, Busan 48434, Republic of Korea; 4 Wellness & Tourism Big Data Research Institute, Kyungsung University, Busan 48434, Republic of Korea

**Keywords:** radar sensors, health monitoring devices, Technology Acceptance Model, perceived credibility, technology anxiety

## Abstract

**Highlights:**

**What are the main findings?**

- Perceived credibility significantly increases users’ perceived usefulness and ease of use of radar-based smart health technologies.
- Technology anxiety reduces intention to use and weakens the effects of core TAM constructs.

**What is the implication of the main finding?**

- Reducing technology anxiety is crucial to improving adoption of sensor-integrated health systems.
- Developers and policymakers should focus on building user trust and tailoring solutions for different user groups, such as students and non-students.

**Abstract:**

Smart health technologies integrating radar sensors enable non-invasive, real-time health monitoring and are central to future-oriented healthcare systems. However, psychological and perceptual barriers may hinder their adoption. This study extends the Technology Acceptance Model (TAM) by incorporating Perceived Credibility (PC) and Technology Anxiety (TA) to examine user acceptance of radar-based health monitoring systems. A quantitative survey was conducted with 222 participants in Binh Duong Smart City, Vietnam. Structural Equation Modeling (SEM) was used to analyze the relationships among variables. Results reveal that PC is significantly associated with Perceived Usefulness (PU) and Perceived Ease of Use (PEOU), which subsequently affect Attitude Toward Use (ATU) and Intention to Use (ITU). Technology Anxiety acts as a psychological barrier, moderating the impact of PC and PEOU on PU, and negatively influencing ATU and ITU. Furthermore, students reported lower PEOU and ITU compared to non-students, suggesting subgroup-specific challenges in adopting sensor-based health systems. These findings enhance understanding of psychological dynamics in the adoption of sensor-integrated health technologies and provide practical implications for designing user-centered smart health solutions that build trust and reduce anxiety.

## 1. Introduction

In today’s age of advanced science and technology, non-invasive monitoring devices have revolutionized healthcare by offering seamless, real-time tracking of vital signs, ensuring early detection and management of health conditions. These cutting-edge technologies provide a comfortable and safe alternative to traditional methods, making quality healthcare more accessible and efficient [1].

Radar sensors stand out among these technologies for their ability to monitor key health indicators such as heart rate and respiration remotely, thus promoting comfort and convenience for both patients and healthcare providers. The Non-Invasive Monitoring Device Market, valued at USD 21.5 billion in 2024, is projected to grow at a compound annual growth rate (CAGR) of 7%, reaching USD 36.39 billion by 2031 [2]. This growth is largely driven by the demand for remote patient monitoring and the rising prevalence of chronic diseases, reinforcing the critical role of these technologies in modern healthcare systems [3].

Despite the technological advancements and potential benefits, the successful adoption of these technologies is not guaranteed. The acceptance and integration of new healthcare devices are largely influenced by user perceptions and trust in the technology. The Technology Acceptance Model (TAM), introduced by Davis in 1989, provides a comprehensive framework for understanding the factors influencing technology adoption [4]. According to TAM, two primary determinants, Perceived Ease of Use (PEOU) and Perceived Usefulness (PU), affect users’ attitudes and intentions to adopt new technologies [4,5]. By exploring key determinants of technology acceptance, TAM offers valuable insights into the intention to use of users, which is essential for the successful integration of new devices in healthcare settings [5,6]. Additionally, in healthcare, where trust and reliability are paramount, additional factors such as Perceived Credibility (PC) have emerged as critical determinants of technology acceptance. When users perceive a technology as credible, they are more likely to trust its usefulness and ease of use, ultimately influencing their decision to adopt the technology [7].

In addition, recent research has also illuminated the relevance of TAM constructs, such as perceived ease of use, usefulness, and credibility, for understanding students’ adoption of information technologies [8]. However, to our knowledge, no study has empirically examined how these perceptions might differ between students and non-students, nor has it focused specifically on health-relating smart technologies. Our study thus extends this prior work by applying an expanded TAM framework, with Perceived Credibility (PC) and Technology Anxiety (TA), to a radar-based smart care system, and by including group comparisons that highlight the distinct experiences of student users, a step that strengthens relevance to the international student research agenda.

Technology Anxiety, defined as the apprehension or fear users feel when interacting with new technologies has been identified as a significant barrier to the acceptance and effective use of healthcare technologies [9]. While technology anxiety can vary across individuals, it is particularly relevant in healthcare settings, where anxiety can reduce users’ willingness to engage with new monitoring devices, even when those devices are user-friendly or perceived as useful. Research shows that TA can negatively impact users’ attitudes toward technology, undermining the positive effects of perceived ease of use and perceived usefulness on the intention to adopt new devices [10].

This research focuses on exploring the role of Perceived Credibility (PC) in shaping user perceptions of radar sensors for health monitoring in the context of Binh Duong Smart City, with particular attention to how Technology Anxiety (TA) moderates key relationships within the Technology Acceptance Model (TAM). The inclusion of TA provides deeper insight into the psychological barriers that may inhibit the acceptance of non-invasive monitoring technologies, even in environments that prioritize technological innovation like smart cities. By analyzing how TA is associated with the relationships between PC, Perceived Usefulness (PU), Perceived Ease of Use (PEOU), and Attitude Toward Use (ATU), this study offers a nuanced understanding of the factors that shape technology adoption in healthcare settings.

In addition to the overall analysis, this study further investigates differences between students and non-students, as students represented a substantial proportion of the respondents. This subgroup analysis highlights how students, particularly international students, may experience distinct levels of technology anxiety and perceptions of ease of use and intention to adopt. Such findings expand the implications of the research, contributing not only to the discourse on smart city healthcare technologies but also to ongoing conversations about student well-being and access to innovation in international education.

## 2. Literature Review

### 2.1. Technology Acceptance in Healthcare

The adoption of new technologies in healthcare has become a critical area of study, especially as the industry increasingly embraces digital and patient-centered models of care. Various theoretical frameworks have been developed to understand the factors influencing technology adoption, with the Technology Acceptance Model (TAM) being one of the most influential. Introduced by Davis in 1989, TAM is widely recognized for its ability to predict and explain user acceptance of technology across various domains [4]. The model posits that perceived ease of use (PEOU) and perceived usefulness (PU) are the primary determinants of users’ intentions to adopt and use a technology. PEOU refers to the degree to which a user believes that using a particular technology will be free of effort, while PU reflects the degree to which the user believes that the technology will enhance their performance. These two factors collectively shape users’ attitudes toward the technology, influencing their intention to use and eventual usage [4,6,11].

Over recent years, TAM has been expanded and adapted to various healthcare contexts, where the adoption of new technologies can significantly impact patient outcomes and the efficiency of healthcare delivery. Researchers have applied TAM to a wide range of healthcare technologies, from electronic health records (EHRs) and telemedicine to wearable health devices and decision support systems. For instance, Alaiad and Zhou (2017) examined the adoption of home healthcare robots using an extended TAM, demonstrating that perceived usefulness and ease of use were significant predictors of acceptance [12]. Similarly, a study conducted on the adoption of Personal Health Records (PHR) systems in Saudi Arabia utilized an extended TAM to investigate how self-determination in health management influences individuals’ intention to adopt PHRs. The study found that perceived ease of use (PEOU), perceived usefulness (PU), and security were major factors influencing the intention to use PHR systems. Additionally, privacy was found to moderate the relationship between PEOU and intention to use, while usability moderated the relationships between PEOU, PU, and intention to use. This study provides critical insights into the factors driving the adoption of PHR systems and highlights the importance of addressing privacy and usability concerns in the implementation of such technologies [13]. These studies highlight the versatility and relevance of TAM in contemporary healthcare, making it a foundational model for understanding how new medical technologies are accepted and integrated into practice.

### 2.2. Perceived Credibility and the Adoption in Healthcare

Perceived credibility (PC) is a critical factor in the adoption of healthcare technologies, influencing users’ trust in the technology and their willingness to engage with it. In the healthcare context, where accuracy, reliability, and security of information are paramount, perceived credibility significantly impacts whether a new technology will be accepted by healthcare professionals and patients. For example, research has shown that users are more likely to adopt mobile health (mHealth) services if they perceive the platforms as credible and trustworthy, which is crucial for ensuring that patients feel comfortable sharing sensitive health data and relying on the technology for critical health decisions [14]. Additionally, the role of perceived credibility has been highlighted in examining the adoption of social media by healthcare professionals, where it was found to significantly is associated with the perceived usefulness of the technology, ultimately affecting their usage behavior [7]. In particular, the trust that users place in these technologies can drive their intention to adopt and use them regularly, making perceived credibility a key determinant in the successful implementation of healthcare innovations [15].

The Technology Acceptance Model (TAM) has been extensively used to understand the adoption of healthcare technologies, with a focus on perceived ease of use (PEOU) and perceived usefulness (PU) as key determinants of technology acceptance. Recent studies have expanded TAM to include factors such as perceived credibility, highlighting its importance in the adoption process. For instance, research on the adoption of electronic health records (EHRs) has found that perceived credibility, including aspects such as data security and system reliability, significantly influences healthcare professionals’ perceptions of a technology’s usefulness and ease of use [5]. Moreover, studies integrating TAM with other behavioral models, such as the Theory of Planned Behavior (TPB), demonstrate that perceived credibility can enhance users’ trust in mobile medical platforms, thereby increasing their willingness to adopt and consistently use these technologies [15]. These findings underscore the critical role of perceived credibility in the successful adoption and integration of healthcare technologies.

### 2.3. Technology Anxiety and Technology Acceptance in Healthcare

Technology Anxiety refers to the apprehension or fear individuals experience when faced with new or unfamiliar technologies. This psychological phenomenon has been widely studied across various domains, particularly in the context of technology adoption, where anxiety can significantly impede user acceptance. In the healthcare sector, where the successful integration of new technologies is vital for improving patient outcomes and enhancing service delivery, TA presents a unique challenge. Users who experience high levels of anxiety are less likely to adopt healthcare technologies, even when those technologies demonstrate significant benefits such as increased accuracy, convenience, and efficiency [10].

In healthcare, where patients and professionals alike rely on accurate, efficient, and secure technologies, Technology Anxiety can reduce the perceived usefulness of such systems. A study by Tsai et al. (2019) on telehealth services revealed that technology anxiety serves as a significant inhibitor to the adoption of such services, regardless of their perceived usefulness and ease of use, thus negatively affecting user intention to adopt these systems [16]. This is particularly problematic in healthcare, where non-adoption can directly affect patient care. For example, users who feel anxious about learning or using radar sensors for health monitoring may fail to recognize the usefulness of such devices, even though they offer significant benefits like non-invasive monitoring and real-time data collection.

### 2.4. Students and Non-Students in Technology Adoption

Students represent an important population in research on technology adoption because their experiences are shaped not only by digital literacy but also by contextual and cultural challenges. While younger individuals are frequently labeled as digital natives, evidence suggests that their adoption of new technologies is associated by psychological factors such as technology anxiety (TA) and perceived credibility (PC), especially when technologies are linked to health and well-being [17]. For students, these challenges are often amplified. Cultural adjustment, language barriers, and unfamiliarity with host-country systems can negatively affect their perceived ease of use (PEOU) and perceived usefulness (PU) of innovative tools [18]. Recent studies show that international students’ confidence in using digital resources is closely tied to institutional support and intercultural competence, highlighting that a lack of support can make the encounter with advanced technology [19].

Similarly, research on generative AI adoption among students demonstrates that constructs such as trust, ease of use, and usefulness play a decisive role in shaping adoption intentions, underscoring the continued relevance of the TAM framework in student populations [20]. These findings collectively indicate that the Technology Acceptance Model (TAM) may operate differently for students, especially in high-stakes domains such as health technology. Extending TAM with constructs like PC and TA provides a more nuanced lens to capture these dynamics. Moreover, by comparing students and non-students within this study, we highlight subgroup-specific differences that contribute to broader conversations in international education about equity, access, and student well-being.

## 3. Methodology

### 3.1. Sample

The study involved 222 participants residing or working in Binh Duong Smart City. A convenience sampling approach was adopted to enable efficient data collection from an accessible population while still ensuring diversity of responses. The online survey link was shared through existing networks in local offices, university communities, and Smart City working groups. Individuals who voluntarily agreed to participate and were able to complete the survey online were included. There were no additional screening procedures, and participation was open to anyone within the outreach channels.

Although convenience sampling may introduce bias due to its non-random nature, it remains a widely used method in exploratory studies where feasibility and participant accessibility are central considerations [21]. The sample size of 222 is considered adequate for Structural Equation Modeling (SEM), as a minimum of 200 participants is generally recommended to ensure stable and reliable parameter estimates [22]. This sample is therefore sufficient to examine the extended Technology Acceptance Model (TAM) in the context of smart city health monitoring, while also allowing subgroup comparisons between students and non-students to provide further insight into differential adoption patterns.

### 3.2. Radar-Based Sensor Description

This study employed a ceiling-mounted radar-based sensor for non-contact health monitoring. The sensor, model CIAZR00K0N00, is manufactured in Korea and designed for indoor use, particularly in bedroom environments. It was installed approximately 170–200 cm above the floor to optimize signal reception directly over the subject.

The device measures 76 × 76 × 12 mm and operates effectively within a detection range of 1.7 m (width) × 2.0 m (length). It uses radar signals to continuously monitor respiratory rate, heart rate, and human movement without requiring any physical contact. When placed on the ceiling, the sensor defines a 2D detection area along the X and Y axes, allowing precise localization of subjects within a Region of Interest (ROI).

**Figure 1:**
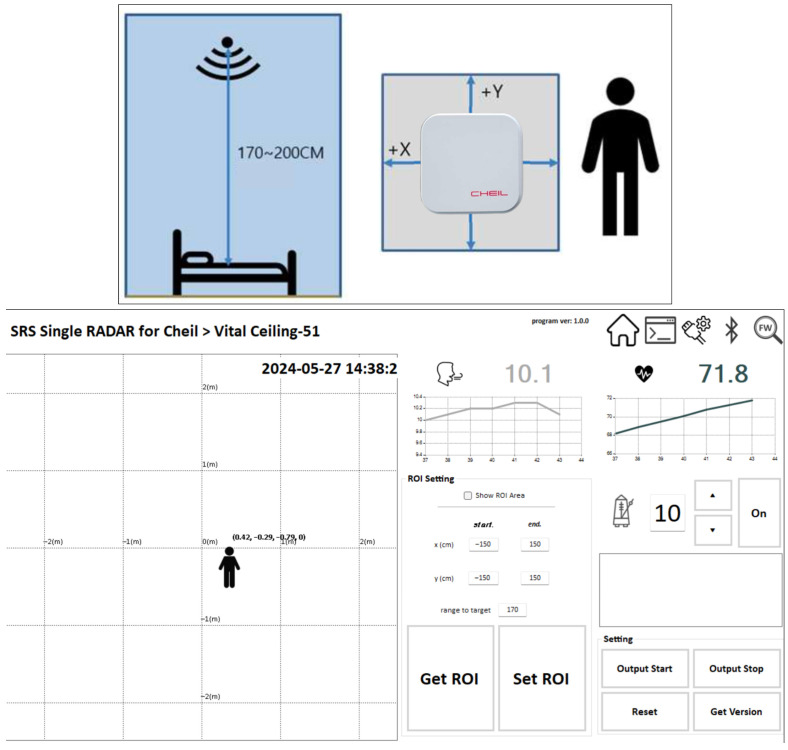
Deployment and operation of the radar sensor used in this study.

Data collected by the sensor are transmitted to a monitoring software platform (Cheil Radar Viewer), as illustrated in Figure 1. The interface displays real-time metrics, including respiration (e.g., 10.1 bpm) and heart rate (e.g., 71.8 bpm), as well as spatial coordinates of the target. The system supports ROI customization, allowing researchers to focus on specific detection zones. This radar-based system was demonstrated and tested in the Binh Duong Smart City context to simulate real-world implementation of smart health monitoring.

### 3.3. Data Collection

Data will be collected using a structured questionnaire designed to measure the key constructs. Table 1 provides a comprehensive overview of each construct measured in the survey on the intention to use radar sensors in a smart care system. It details the number of items, measurement scales, purposes, and explanations for each construct, along with relevant academic references that support the constructs within the framework of the TAM.

Several procedural controls are implemented during the survey design and data collection process. The questionnaire, using 5-point Likert scale, ensures respondent anonymity and voluntary participation, avoiding any personal identifiers. The survey items are adapted from established constructs used in prior validated models, with standardized measurement scales. Detailed information on item sources and reliability is presented in Table 1. The items are randomized to prevent response pattern bias, and neutral wording is employed to reduce social desirability effects. Importantly, no physical or physiological measurements were conducted on participants. The study relied solely on self-reported survey responses, and no sensor data were collected directly from human subjects, thereby minimizing ethical or privacy concerns.

### 3.4. Research Model

This study adopts the Technology Acceptance Model (TAM) as the theoretical foundation to examine factors influencing the adoption of radar sensors in smart healthcare systems. Within the TAM framework, Perceived Ease of Use (PEOU), Perceived Usefulness (PU), and Attitude Toward Use (ATU) are central determinants of Intention to Use (ITU). To extend TAM to the healthcare context, Perceived Credibility (PC) is incorporated as a key construct, given the critical importance of trust and reliability in medical technologies.

In addition, Technology Anxiety (TA) is introduced as a moderating factor. TA captures the apprehension or discomfort individuals experience when interacting with new technologies, which can weaken positive relationships among TAM constructs. For example, even if users recognize a technology as credible, useful, or easy to use, high levels of TA may still reduce their willingness to adopt it.

**Figure 2:**
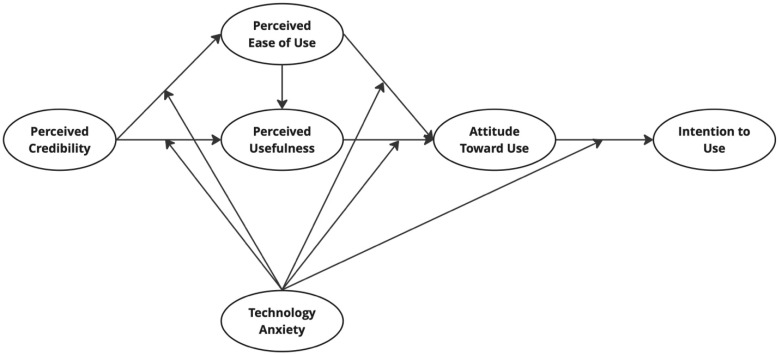
Proposed research model.

By integrating PC and TA into TAM, the proposed research model (Figure 2) provides a comprehensive framework to investigate how trust, ease of use, usefulness, and anxiety jointly influence adoption intentions. This extended model enables the study to capture not only the direct effects among constructs but also the moderating role of TA, thereby offering a more nuanced understanding of radar sensor acceptance in healthcare and smart city contexts.

### 3.5. Research Analysis

The data analysis was conducted using SPSS 28 and SmartPLS 4.0, also the authors used ChatGPT 4.0 to assist in language editing and restructuring. The authors take full responsibility for the content. First, the reliability of each construct was evaluated through Cronbach’s alpha, with the threshold of 0.70 used as a benchmark for acceptable internal consistency. Convergent validity was then assessed by examining the factor loadings of all measurement items, ensuring that each loading exceeded the recommended threshold of 0.70. In addition, the average variance extracted (AVE) was calculated for each construct, with values greater than 0.50 indicating adequate convergent validity [25].

After the measurement model was confirmed, the structural model was analyzed in SmartPLS using a bootstrapping procedure to estimate path coefficients and test the proposed research model. This approach allowed for the evaluation of both direct effects within the extended Technology Acceptance Model (TAM) and the moderating role of Technology Anxiety (TA). The two-stage approach is applied to examine the moderating role of Technology Anxiety (TA), following best practices in PLS-SEM for reflective models.

To further explore subgroup differences, an independent samples *t*-test was conducted in SPSS, comparing students (coded as 1) and non-students (coded as 0) across the main constructs, including Perceived Credibility (PC), Technology Anxiety (TA), Perceived Ease of Use (PEOU), Perceived Usefulness (PU), Attitude Toward Use (ATU), and Intention to Use (ITU). Following prior research that employed independent samples *t*-tests to compare group differences in technology adoption [26], by examining whether students and non-students differed significantly across key TAM constructs. This additional analysis provided insights into whether user background influences perceptions and adoption intentions toward radar-based healthcare technologies.

## 4. Results

**Figure 3:**
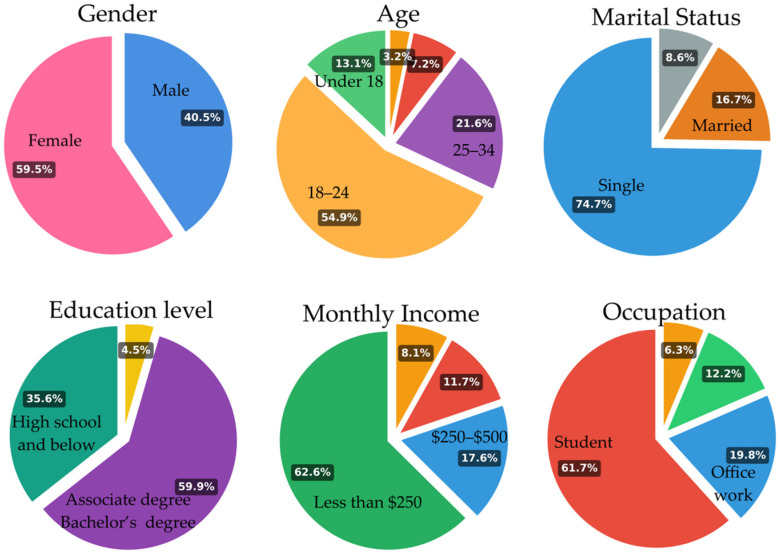
Demographics of respondents (n = 222).

A total of 222 valid responses were collected for this study (see Figure 3). Regarding gender, the sample included 59.5% female and 40.5% male participants. In terms of age distribution, the majority (54.9%) of respondents were between 18 and 24 years old, followed by 21.6% aged 25–34, and 13.1% were under 18 years old, indicating a largely young population. A smaller portion of respondents fell into the 35–44 (7.2%) and 45 and over (3.2%) categories.

In terms of marital status, 74.7% of participants were single, while 16.7% were married, and 8.6% chose “other,” reflecting a predominance of younger and unmarried individuals. Regarding educational background, 59.9% had completed a Bachelor’s or Associate degree, while 35.6% reported having completed high school or below, and only a small portion held a graduate degree.

With respect to income, 62.6% of respondents reported earning less than $250 per month, indicating limited financial capacity among a majority of participants. The remaining respondents reported monthly incomes between $250–$500 (17.6%), $500–$1000 (11.7%), and above $1000 (8.1%).

Finally, in terms of occupation, 61.7% of the sample were students, while 19.8% were office workers and 12.2% were officials or civil servants. These results confirm that the sample was largely composed of young, educated individuals, many of whom are still in school or in the early stages of their careers.

Table 2 presents the reliability and validity assessment results for the constructs employed in the extended Technology Acceptance Model (TAM), which includes the additional variables Perceived Credibility (PC) and Technology Anxiety (TA). All constructs exhibit strong internal consistency, as evidenced by Cronbach’s Alpha values exceeding 0.87 and Composite Reliability (CR) values greater than 0.90, surpassing the commonly accepted thresholds and confirming the reliability of the measurement scales [25].

Furthermore, all standardized factor loadings for individual measurement items were above 0.70, indicating that each item contributes significantly to its respective latent construct. The Average Variance Extracted (AVE) values for all constructs were also above the recommended minimum of 0.50, thereby supporting the convergent validity of the constructs [27].

Discriminant validity was assessed using the Fornell–Larcker criterion. As shown in Table 3, the square root of the Average Variance Extracted (AVE) for each construct (presented on the diagonal in bold) was greater than its correlations with any other construct. This indicates that each latent variable shares more variance with its own indicators than with other constructs in the model, thereby satisfying the Fornell–Larcker criterion and supporting discriminant validity [25].

To assess the quality of the structural model, several key diagnostic metrics were examined, including R^2^, Q^2^, f^2^ effect sizes, and multicollinearity statistics (VIF). R^2^ values for all endogenous constructs indicate substantial explanatory power [25]. Attitude Toward Use (ATU) = 0.752, Intention to Use (ITU) = 0.724, Perceived Usefulness (PU) = 0.749, and Perceived Ease of Use (PEOU) = 0.541. These values suggest that the model explains a large proportion of variance in user attitudes and intentions, consistent with prior TAM-based studies.

Q^2^ values, obtained via blindfolding procedures with cross-validated redundancy, were all above zero, confirming the predictive relevance of the model [25]. PU = 0.654, ATU = 0.618, ITU = 0.591, and PEOU = 0.400. These results suggest that the model not only fits the data but also has strong out-of-sample predictive accuracy. Then, f^2^ effect sizes were computed for each path to assess local impact. Strong effects were observed for ATU → ITU (f^2^ = 2.130), PC → PEOU (f^2^ = 0.849), and PU → ATU (f^2^ = 0.510). Moderate effects were found for PEOU → PU (f^2^ = 0.374), and smaller effects were noted for PEOU → ATU (f^2^ = 0.119) and TA moderation paths (ranging from 0.000 to 0.037). These results support the relative importance of core TAM constructs while identifying more modest contributions from moderation effects.

Multicollinearity was assessed using both outer and inner VIF values. All VIFs were below the recommended threshold of 5.0 for outer indicators, except for a few items (PU1 = 6.133, PU3 = 6.034, PC2 = 5.680, PC4 = 5.177), which slightly exceeded this threshold. Inner VIF values for structural paths were all below 3.0, indicating no serious multicollinearity among latent constructs. Thus, multicollinearity is not a major concern in this model [25].

The model fit (Table 4) indices show that both the Saturated Model and Estimated Model demonstrate acceptable. The SRMR values (0.059 for the Saturated Model, 0.085 for the Estimated Model) are within acceptable limits. The d_ULS and d_G values are lower for the Saturated Model, indicating a better fit. The Chi-Square for the Estimated Model (936.842) is marginally better than the Saturated Model (951.898), suggesting slight improvement. Both models show reasonable NFI values (0.850 and 0.853), though still below the ideal 0.90 threshold, indicating acceptable model fit.

The structural model is evaluated using path analysis to test the hypothesized relationships among constructs in the extended TAM framework. All direct paths between the core variables are found to be statistically significant (Table 5).

Specifically, the structural model shows strong support for the core pathways of the Technology Acceptance Model (TAM). Perceived Credibility (PC) exhibite a significant positive effect on both Perceived Usefulness (PU) (*β* = 0.458, SD = 0.094, *t* = 4.875, *p* < 0.001) and Perceived Ease of Use (PEOU) (*β* = 0.598, SD = 0.055, *t* = 10.877, *p* < 0.001). PEOU is significantly associated with PU (*β* = 0.437, SD = 0.084, *t* = 5.233, *p* < 0.001) and Attitude Toward Use (ATU) (*β* = 0.269, SD = 0.092, *t* = 2.930, *p* = 0.003). PU is a strong predictor of ATU (*β* = 0.536, SD = 0.112, *t* = 4.778, *p* < 0.001), and ATU has the strongest effect on Intention to Use (ITU) (*β* = 0.850, SD = 0.035, *t* = 24.535, *p* < 0.001), confirming the proposed TAM structure.

Moderation analysis reveals three statistically significant interactions involving Technology Anxiety (TA). TA negatively moderate the relationship between PC and PEOU (*β* = −0.096, SD = 0.031, *t* = 3.094, *p* = 0.002), as well as between PU and ATU (*β* = −0.332, SD = 0.108, *t* = 3.063, *p* = 0.002), indicating that higher levels of anxiety weaken these associations. In contrast, TA positively moderate the path from PEOU to ATU (*β* = 0.240, SD = 0.101, *t* = 2.385, *p* = 0.017), suggesting that ease of use becomes even more important in shaping attitudes when anxiety is high. Meanwhile, the interaction effects of TA on PC to PU (*β* = −0.034, *p* = 0.090) and ATU to ITU (*β* = 0.024, *p* = 0.267) are not statistically significant, indicating limited or no moderating influence along these paths.

To further explore the moderating role of Technology Anxiety (TA), a simple slope analysis was conducted at three levels of TA: low (−1SD), average, and high (+1SD). As shown in Table 6, TA significantly moderates three structural paths. First, the effect of Perceived Credibility (PC) on Perceived Ease of Use (PEOU) weakens as TA increases: *β* = 0.704 at low TA, *β* = 0.610 at average TA, and *β* = 0.516 at high TA. Similarly, the path from Perceived Usefulness (PU) to Attitude Toward Use (ATU) shows a decreasing effect under high TA (*β* = 0.550), compared to low TA (*β* = 0.776). In contrast, TA strengthens the relationship between PEOU and ATU, with the slope increasing from *β* = 0.539 (high TA) to β = 0.754 (low TA), suggesting that ease of use becomes more influential when anxiety is lower. All interactions are statistically significant (*p* < 0.001), highlighting the conditional nature of these relationships based on user anxiety levels.

In addition to the primary analysis, we examine potential group differences between students and non-students in their perceptions and intention to use toward the radar-based smart care system. Independent samples *t*-tests are conducted on six key constructs: Perceived Credibility (PC), Technology Anxiety (TA), Perceived Ease of Use (PEOU), Perceived Usefulness (PU), Attitude Toward Use (ATU), and Intention to Use (ITU). Descriptive and inferential results are summarize in Table 7.

The results indicated statistically significant group differences in two constructs. Non-students reported higher Perceived Ease of Use (PEOU; M = 3.51, SD = 0.67) than students (M = 3.27, SD = 0.65), *t*(220) = −2.65, *p* = 0.009. Similarly, Intention to Use (ITU) was higher among non-students (M = 3.58, SD = 0.74) compared to students (M = 3.35, SD = 0.74), *t*(220) = −2.23, *p* = 0.027. No statistically significant differences were found for Perceived Usefulness (PU), Perceived Credibility (PC), Attitude Toward Use (ATU), or Technology Anxiety (TA), although differences in PC and ATU approached significance (*p* = 0.062 and *p* = 0.055, respectively).

## 5. Discussion

### 5.1. Summary of Key Findings

This study examined the adoption of radar sensors in healthcare settings by extending the Technology Acceptance Model (TAM) to include Perceived Credibility (PC) and Technology Anxiety (TA). The results confirm the robustness of core TAM constructs, including Perceived Ease of Use (PEOU), Perceived Usefulness (PU), Attitude Toward Use (ATU), and Intention to Use (ITU), in predicting technology acceptance. The adoption of AI-based tools in healthcare remains limited unless providers and patients perceive clear transparency, empathy, and alignment with existing clinical workflows [28]. Also, the influence of perceived credibility on user intention is consistent with prior research, which highlights that trust in information sources and digital interfaces significantly enhances user engagement and acceptance [29].

Notably, PC is found to be significantly associated with both PU and PEOU, indicating that users who perceive radar sensors as credible are more likely to view them as beneficial and easy to use. This reinforces earlier findings on the centrality of trust and security perceptions in healthcare technology adoption [7,15].

In line with classical TAM theory, PEOU positively impacts PU and ATU, suggesting that systems perceived as easy to navigate enhance both perceived benefits and favorable attitudes, which is consistent with prior work [13]. PU also has a strong positive effect on ATU, reaffirming that usefulness perceptions play a critical role in shaping user attitudes [30].

The strongest relationship observe in the model was between ATU and ITU, indicating that user attitudes are the most influential driver of intention to use. This finding aligns with the broader TAM literature emphasizing the importance of fostering positive attitudes for sustainable adoption [10].

### 5.2. Role of Technology Anxiety (TA) as a Moderator

The moderation analysis revealed that Technology Anxiety (TA) significantly interacted with three structural paths in the model. First, TA negatively moderated the relationship between Perceived Credibility (PC) and Perceived Ease of Use (PEOU), suggesting that as anxiety increases, the association between credibility and ease of use becomes weaker. Second, TA also moderated the link between Perceived Usefulness (PU) and Attitude Toward Use (ATU), indicating that users experiencing higher anxiety levels may form less favorable attitudes even when they perceive the system as useful. Interestingly, TA positively moderated the path between PEOU and ATU, implying that ease of use becomes more influential in shaping user attitudes under conditions of heightened anxiety.

In contrast, two moderating effects were not statistically significant. The interaction between TA and PC on PU and between TA and ATU on ITU did not reach conventional levels of significance. These non-significant interactions suggest that TA may not meaningfully influence the strength of these particular relationships within this model. Overall, the results highlight the nuanced role of anxiety in modulating user perceptions and attitudes, particularly during early-stage evaluations of unfamiliar healthcare technology. Moreover, prior research has emphasized that user concerns about technology complexity and trustworthiness play a crucial role in shaping healthcare technology adoption, particularly among patients and providers with limited digital literacy [31].

The negative moderation of TA on the relationship between perceived credibility and perceived ease of use, as well as between perceived usefulness and attitude toward use, indicates that these associations become weaker when users experience higher levels of anxiety [32]. In contrast, the positive moderation of TA on the relationship between perceived ease of use and attitude toward use suggests that ease of use becomes more important in shaping attitudes under conditions of high anxiety. These findings highlight the importance of addressing user anxiety when designing and implementing healthcare technologies [33].

### 5.3. Group Differences: Student vs. Non-Student Users

The demographic subgroup analysis revealed significant differences between students and non-students. Students reported significantly lower levels of PEOU and ITU, suggesting that younger users may struggle more with radar-based systems, potentially due to less exposure to health-related technologies and lower perceived urgency. These users may lack the confidence or motivation to explore complex health systems, especially if they perceive them as irrelevant to their current life stage.

Interestingly, although differences in PC and ATU were not statistically significant, they approached significance, implying potential trends that warrant further exploration. These near-threshold results suggest that students may also have lower trust and less favorable attitudes, albeit not strongly enough to reach significance in this study. Tailoring technology interfaces and communication strategies to match the digital expectations and needs of student users may enhance acceptance in this group.

## 6. Implication

### 6.1. Theoretical Imlication

This study extends the Technology Acceptance Model (TAM) by integrating Perceived Credibility (PC) and Technology Anxiety (TA), offering new theoretical insights into technology adoption. The inclusion of PC highlights how trust in system reliability and data security enhances user confidence, which directly fosters intention to use. Such findings resonate with prior work in sensor-based healthcare monitoring, where credibility and transparency were fundamental in ensuring long-term adherence [34]. By situating PC as a critical construct, this study advances TAM toward a more trust-centered explanatory framework.

Another theoretical contribution lies in the incorporation of TA, which represents the emotional and affective barriers to technology adoption. The results indicate that TA is negatively associated with intention, particularly for non-student groups with lower technological familiarity. This complements earlier studies in non-contact sensing environments, where user concerns regarding complexity and signal reliability hindered engagement [35]. By embedding TA into TAM, our research underscores the importance of considering psychological barriers alongside cognitive determinants such as usefulness and ease of use.

Finally, the comparative analysis between students and non-students demonstrates that TAM extensions are contingent upon demographic and experiential contexts. The moderating effect of user groups implies that adoption models cannot be generalized across heterogeneous populations. Consequently, the present research enriches TAM theory by integrating contextual and affective variables, thereby broadening its explanatory power in the domain of intelligent sensing technologies.

### 6.2. Managerial Implications

From a managerial perspective, this study provides actionable insights for the deployment of sensor-enabled technologies. First, managers should prioritize system credibility, emphasizing robust data protection, transparent communication, and reliable system performance. Similar to evidence in gait analysis, where reliable sensor-based systems fostered trust in real-world applications, organizations should highlight these features to enhance user engagement and compliance [36,37].

Second, strategies must focus on mitigating technology anxiety, particularly among non-student or less technologically literate populations. Training initiatives, intuitive user interfaces, and user-centered onboarding processes can substantially reduce apprehension. These approaches mirror findings in intelligent healthcare classification systems, where usability and accessibility improvements enhanced adoption among diverse stakeholders [38]. Thus, investment in supportive infrastructures is essential for overcoming anxiety-driven resistance.

Beyond managerial considerations, the findings provide guidance for policymakers and health technology developers aiming to promote equitable smart health adoption. Public health authorities should develop policies that enhance citizens’ digital literacy and awareness of data privacy, thereby fostering trust in emerging monitoring systems [39]. Educational programs targeting specific user groups, such as younger individuals or those with limited technological familiarity, can help reduce technology anxiety through structured training and transparent communication about system functionality and data handling. Integrating these initiatives into broader smart health policy frameworks would not only strengthen public acceptance but also ensure that sensor-based innovations contribute to more inclusive and sustainable healthcare ecosystems [40,41].

## 7. Limitations and Future Research

Although this study provides novel insights into technology acceptance by extending TAM with Perceived Credibility and Technology Anxiety, several limitations should be acknowledged. First, the dataset was limited to a cross-sectional survey, which constrains causal inference and may not fully capture the dynamic evolution of user perceptions over time [42,43]. Moreover, the use of convenience sampling may have limited the representativeness of the sample, as participation was based on accessibility rather than random selection. This could lead to potential sampling bias, particularly given the relatively high proportion of student respondents. In addition, the reliance on self-reported data introduces the possibility of response bias and perceptual inaccuracy, as participants’ answers may reflect subjective evaluations rather than actual behavior. Although procedural controls such as anonymity and item randomization were implemented to mitigate these effects, future studies should consider triangulating self-reported measures with behavioral or longitudinal data to strengthen methodological robustness.

Second, the sample was restricted to student and non-student groups within a specific cultural and national context. While the multi-group analysis highlighted meaningful differences, the findings may not be generalizable across broader populations, industries, or geographic regions. Additionally, subgroup comparisons between students and non-students were based on independent-sample *t*-tests without prior measurement invariance testing. This approach is exploratory and should be interpreted cautiously due to potential construct nonequivalence.

Third, the study employed self-reported measures to assess constructs such as credibility and anxiety, which may be subject to bias. Future research could incorporate objective behavioral data, such as actual system usage logs or physiological measures of anxiety, to complement perceptual assessments. Moreover, integrating additional external variables (e.g., perceived risk, trust in AI, or social influence) may further enrich the model. By addressing these limitations, subsequent studies can advance theoretical robustness and enhance the practical applicability of TAM in sensor-enabled and intelligent technology domains.

Lastly, despite the strength of the structural model, several limitations warrant consideration. The sample was skewed toward university students, which may affect the generalizability of the findings to broader populations, including older users or healthcare professionals. Moreover, the study was context-specific to Binh Duong Smart City, whose unique infrastructural and technological landscape may not reflect conditions elsewhere. Interpretation of findings should also consider the marginal nature of certain model-fit indices, suggesting that caution is needed when assessing model robustness. Although this study was exempt from formal institutional review due to the anonymous and minimal-risk nature of data collection, this exemption is acknowledged for transparency. Practically, the results suggest that enhancing credibility and perceived ease of use can support adoption, particularly when addressing user anxiety through interface design and targeted onboarding. For successful deployment of radar sensing in healthcare, localized implementation strategies, such as co-design with end-users and awareness initiatives, should be prioritized to ensure trust, usability, and contextual fit.

## Figures and Tables

**Table 1:** Detailed explanation of constructs measured in data collection.

Construct	Items	References
Perceived Ease of Use (PEOU)	PEOU1: Using the radar sensor would be easy for me	[4,23]
	PEOU2: Learning to operate the radar sensor would be easy for me	
	PEOU3: I think the radar sensor would be user-friendly	
	PEOU4: The radar sensor would require little effort to use	
Perceived Usefulness (PU)	PU1: Using the radar sensor would enhance my health monitoring capabilities	[4,23]
	PU2: The radar sensor would improve the effectiveness of my health management	
	PU3: I think the radar sensor would be useful for my health needs	
	PU4: Using the radar sensor would provide me with valuable health information	
Perceived Credibility (PC)	PC1: I would trust the information provided by the radar sensor	[7,15]
	PC2: I would consider the radar sensor to be reliable	
	PC3: I would feel confident in the accuracy of the radar sensor	
	PC4: I think the radar sensor is a trustworthy device	
Attitude Toward Use (ATU)	ATU1: I would have a positive attitude towards using the radar sensor	[4]
	ATU2: I think using the radar sensor is a good idea	
	ATU3: I would enjoy using the radar sensor	
	ATU4: I think the radar sensor would be beneficial for my health	
Intention to Use (ITU)	ITU1: I intend to use the radar sensor regularly if available	[4,23]
	ITU2: I plan to use the radar sensor in the future	
	ITU3: I would recommend the radar sensor to others	
	ITU4: I am likely to purchase a radar sensor for personal use	
Technology Anxiety (TA)	TA1: I feel worried about personal information not being kept secure when using new technology	[10,24]
	TA2: I feel apprehensive about using new technologies	
	TA3: Using new technologies makes me nervous	
	TA4: I hesitate to use new technology because it is unfamiliar	

**Table 2:** Factor loading and reliability test.

Construct	Item	Factor Loading	Cronbach’ Alpha	rho_A	Composite Reliability	AVE
PEOU	PEOU1	0.858	0.892	0.893	0.925	0.755
	PEOU2	0.889				
	PEOU3	0.862				
	PEOU4	0.866				
PU	PU1	0.953	0.957	0.957	0.969	0.885
	PU2	0.932				
	PU3	0.952				
	PU4	0.926				
PC	PC1	0.931	0.956	0.956	0.968	0.883
	PC2	0.947				
	PC3	0.941				
	PC4	0.939				
ATU	ATU1	0.926	0.933	0.935	0.952	0.833
	ATU2	0.927				
	ATU3	0.870				
	ATU4	0.928				
ITU	ITU1	0.924	0.930	0.933	0.950	0.826
	ITU2	0.930				
	ITU3	0.902				
	ITU4	0.878				
TA	TA1	0.787	0.873	0.901	0.910	0.718
	TA2	0.911				
	TA3	0.860				
	TA4	0.827				

Note. Perceived Ease of Use (PEOU); Perceived Usefulness (PU); Perceived Credibility (PC); Attitude Toward Use (ATU); Intention to Use (ITU); Technology Anxiety (TA); Average Variance Extracted (AVE).

**Table 3:** Discriminant validity (Fornell-Larcker criterion).

	ATU	ITU	PC	PEOU	PU	TA
ATU	**0.913**					
ITU	0.849	**0.909**				
PC	0.868	0.792	**0.940**			
PEOU	0.787	0.694	0.724	**0.869**		
PU	0.847	0.774	0.806	0.800	**0.941**	
TA	0.380	0.370	0.370	0.388	0.363	**0.848**

**Table 4:** Model fit.

	Saturated Model	Estimated Model
SRMR	0.059	0.085
d_ULS	1.048	2.144
d_G	0.721	0.837
Chi-Square	951.898	936.842
NFI	0.850	0.853

**Table 5:** Summary of hypotheses testing results.

Path	Path Coefficient (*β*)	SD	*t* Value	*p* Value	
PC → PU	0.458	0.094	4.875	<0.001	Significant
PC → PEOU	0.598	0.055	10.877	<0.001	Significant
PEOU → PU	0.437	0.084	5.233	<0.001	Significant
PEOU → ATU	0.269	0.092	2.930	0.003	Significant
PU → ATU	0.536	0.112	4.778	<0.001	Significant
ATU → ITU	0.850	0.035	24.535	<0.001	Significant
TA moderates PC → PEOU	−0.096	0.031	3.094	0.002	Significant
TA moderates PU → ATU	−0.332	0.108	3.063	0.002	Significant
TA moderates PEOU → ATU	0.240	0.101	2.385	0.017	Significant
TA moderates PC → PU	−0.034	0.020	1.698	0.090	Not Significant
TA moderates ATU → ITU	0.024	0.022	1.110	0.267	Not Significant

Note. Perceived Ease of Use (PEOU); Perceived Usefulness (PU); Perceived Credibility (PC); Attitude Toward Use (ATU); Intention to Use (ITU); Technology Anxiety (TA); Standard Deviation (SD).

**Table 6:** Simple slope analysis of the moderating effect of technology anxiety.

		Estimate (*β*)	SE	Z	*p*
TA moderates PC → PEOU	Average	0.610	0.0503	12.13	<0.001
	Low (−1SD)	0.704	0.0470	14.97	<0.001
	High (+1SD)	0.516	0.0703	7.34	<0.001
TA moderates PU → ATU	Average	0.663	0.0363	18.3	<0.001
	Low (−1SD)	0.776	0.0332	23.4	<0.001
	High (+1SD)	0.550	0.0526	10.4	<0.001
TA moderates PEOU → ATU	Average	0.647	0.0446	14.50	<0.001
	Low (−1SD)	0.754	0.0407	18.52	<0.001
	High (+1SD)	0.539	0.0639	8.43	<0.001

Note. Perceived Ease of Use (PEOU); Perceived Usefulness (PU); Perceived Credibility (PC); Attitude Toward Use (ATU); Technology Anxiety (TA); Standard Error (SE).

**Table 7:** Independent samples *t*-test results comparing students and non-students.

Variable	Group	N	M	SD	*t* (df)	*p*
PEOU	Student	137	3.27	0.65	−2.65 (220)	0.009
	Non-Student	85	3.51	0.67		
PU	Student	137	3.41	0.71	−1.57 (220)	0.118
	Non-Student	85	3.56	0.69		
PC	Student	137	3.29	0.64	−1.88 (220)	0.062
	Non-Student	85	3.47	0.68		
ATU	Student	137	3.36	0.64	−1.93 (220)	0.055
	Non-Student	85	3.53	0.65		
ITU	Student	137	3.35	0.74	−2.23 (220)	0.027
	Non-Student	85	3.58	0.74		
TA	Student	137	3.06	0.81	−1.71 (220)	0.090
	Non-Student	85	3.25	0.75		

Note. M: Mean; SD: Standard Deviation. Independent samples *t*-tests were conducted assuming equal variances based on Levene’s tests.

## Data Availability

The data presented in this study are available on request from the corresponding author. The data are not publicly available due to privacy restrictions.
